# Strain Effect on Electronic Structure and Work Function in α-Fe_2_O_3_ Films

**DOI:** 10.3390/ma10030273

**Published:** 2017-03-09

**Authors:** Li Chen, Changmin Shi, Xiaolong Li, Zhishan Mi, Dongchao Wang, Hongmei Liu, Lijie Qiao

**Affiliations:** 1 Institute of Condensed Matter Physics, Linyi University, Linyi 276000, China; shichangmin@lyu.edu.cn (C.S.); lixiaolong@lyu.edu.cn (X.L.); wdcowdc@126.com (D.W.); Liuhongmei@lyu.edu.cn (H.L.); 2 Corrosion and Protection Center, Key Laboratory for Environmental Fracture (MOE), University of Science and Technology Beijing, Beijing 100083, China; mizhishan@163.com

**Keywords:** α-Fe_2_O_3_ films, strain, band gap, work function, corrosion potential, 71.20.Nr, 71.70.Fk, 73.20.At

## Abstract

We investigate the electronic structure and work function modulation of α-Fe_2_O_3_ films by strain based on the density functional method. We find that the band gap of clean α-Fe_2_O_3_ films is a function of the strain and is influenced significantly by the element termination on the surface. The *p_x_* and *p_y_* orbitals keep close to Fermi level and account for a pronounced narrowing band gap under compressive strain, while unoccupied *d_z_*_2_ orbitals from conduction band minimum draw nearer to Fermi level and are responsible for the pronounced narrowing band gap under tensile strain. The spin polarized surface state, arising from localized dangling-bond states, is insensitive to strain, while the bulk band, especially for *p_z_* orbital, arising from extended Bloch states, is very sensitive to strain, which plays an important role for work function decreasing (increasing) under compressive (tensile) strain in Fe termination films. In particular, the work function in O terminated films is insensitive to strain because *p_z_* orbitals are less sensitive to strain than that of Fe termination films. Our findings confirm that the strain is an effective means to manipulate electronic structures and corrosion potential.

## 1. Introduction

Metals are protected from corrosion in many aqueous environments by the formation of “passive” oxide films that are typically only a few nanometers thick. The transition metal oxides are an important class of functional materials [1] because of their localized *d* electrons [2,3,4]. Among the iron oxides, α-Fe_2_O_3_, hematite, with an experimental band gap of 2.2 eV [5] was identified as a charge transfer semiconductor [2] rather than a Mott–Hubbard insulator because the upper edge of the valence band is dominated by oxygen *p* states. Below the Néel temperature (955 K), α-Fe_2_O_3_ is an antiferromagnetic (AFM) insulator showing weak ferromagnetism above the Morin temperature (260 K), due to a slight canting of the two sublattice magnetizations [6]. The geometric, electronic, and magnetic properties [7,8,9]; carrier effective masses [10]; and other related properties on Fe_2_O_3_ [11] were present in theoretical work.

The technological importance of these films has led to widespread investigation of their structure, chemistry [12] and practical applications such as water splitting [13]. The atomic structure of α-Fe_2_O_3_ is important to passivity, since this often controls its protective properties, where interfacial strain due to lattice mismatch and charge transfer due to chemical heterogeneity between the Fe oxide film and steel are unavoidable. There could be different factors, such as epitaxial strain, structural deformation, molecular orbital hybridization, and interfacial charge transfer, which affect the properties of the film. However, experimental and theoretical analyses suggest strain and charge transfer are two important extrinsic factors to alter the intrinsic properties of ultrathin Fe oxide films grown on a stainless steel (SS). In fact, strain and interfacial engineering have now become a common strategy to tailor a wide range of thin film properties, from crystalline structure [14], growth morphology (especially nanoscale assembly) [15], electronic band structure [16], magnetism [17], and carrier mobility [18] to superconductivity [19].

It is generally known that SS resistance to stress corrosion cracking is poor, which is largely affected by the structure, thickness, and composition of the passive film [20]. The strain is an effective approach to modulate the surface work function. Based on experimental observations and theoretical studies, we know that the work function is closely related to the corrosion potential, the lower work function of material, the lower corrosion potential is and the easier corrosion becomes, i.e., the work function is a sensitive parameter to the corrosion behavior [21]. Strain is expected to affect the fundamental properties of systems, and conversely can be used as an effective mean to tailor the properties of materials for potential applications.

From a theoretical point of view, the main interest in these compounds lies in the role of strong electronic correlations in determining their physical and chemical properties. Motivated by these recent experimental and theoretical studies, we have carried out a systematic study of the electronic properties of α-Fe_2_O_3_ film focusing on the effects of strain, based on first-principles calculations. We find that the band gap of the α-Fe_2_O_3_ film and work function can be tuned by strain. These calculation results allow us to conclude that the electronic structure and work function of α-Fe_2_O_3_ film could be modified by strain when the α-Fe_2_O_3_ film is grown on a SS, which can be very useful for explaining the effect of strain, future experimental studies as well as fundamentally understand the corrosion behavior.

## 2. Methods

**Figure 1:**
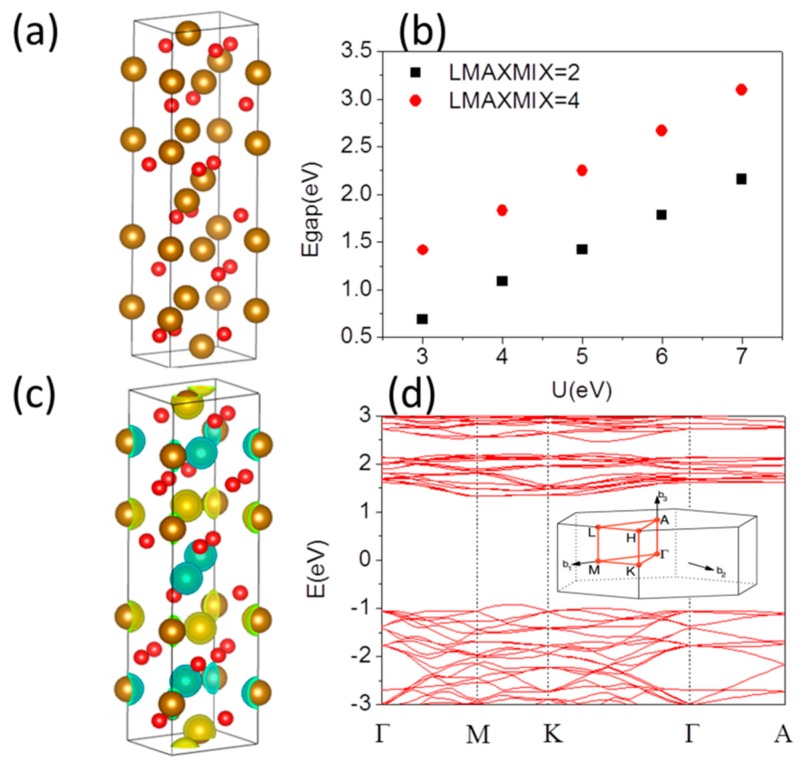
(**a**) The lattice geometry of α-Fe_2_O_3_ bulk from side view, the yellow and red color is marked for Fe and O; respectively; (**b**) band gap of α-Fe_2_O_3_ bulk as a function of U parameter of Coulomb interaction; (**c**) magnetic spin density at isosurface level at 0.2 e/Å^3^ with light yellow and green color for up and down spin, respectively; and (**d**) band structure of α-Fe_2_O_3_ bulk for lattice constant *a* = 4.9158 Å at U = 5 eV with Γ(0.00, 0.00, 0.00), M(0.00, 0.50, 0.00), K(−0.33, 0.67, 0.00), and A(0.00, 0.00, 0.50). The Fermi level is set to zero and band gap is 2.224 eV.

First, as is typical for transition-metal compounds, both GGA and the LDA+U corrections reduce the LDA overbinding problem and increase the equilibrium volume which becomes close to the experimental value. Rohrbach et al. [22] performed a GGA+U calculation for Cr_2_O_3_ and obtained more reasonable results for the band structure. However, this approach is inaccurate for structural and magnetic properties. Mosey et al. [23] obtained better overall agreement with experiment for structural and spectral properties using the spherically averaged LSDA+U method with U − J = 3.2 eV. For α-Fe_2_O_3_, because Fe d bands are strongly correlated, researchers adopted the LSDA+U approach [24,25]. Therefore, we tend to use LDA+U method for α-Fe_2_O_3_ system. The hexagonal lattice of α-Fe_2_O_3_ film is shown in Figure 1a. The electronic band structures and electronic properties were calculated as before [26,27,28,29,30] in the framework of the (Slater exchange [31] plus Vosko–Wilk–Nusair correlation [32]) local density approximation LDA+U using Vienna Ab initio Simulation Package (VASP) package. The energy cutoff of 400 eV was selected to converge the total energy within 1 meV/atom on 5 × 5 × 5 and 5 × 5 × 1 (or 9 × 9 × 1) Monkhorst–Pack k-point mesh for bulk and film, respectively. For the calculation of band structures within the L(S)DA+U approach, it is hence strictly required to increase LMAXMIX (a parameter in VASP code) to 4 (*d* elements). For structural relaxation, all the atoms are allowed to relax until atomic forces are smaller than 0.01 eV/Å. The optimized lattice constant for bulk is *a* = 4.916 Å which is close to experimentally derived structural parameter 5.035 Å [33]. It is expected that different surface Fe and O terminations will also lead to significant differences in the electronic and work function of the systems. We defined the strain percent as (*a* − *a*_0_)/*a*_0_, where *a*_0_ is the experimental lattice constant without strain, and *a* is the lattice constant under compressive or tensile strain. The polar (0001) surface has been modeled by symmetric slabs containing 1–3 hexagonal unit cell layers for the Fe and O terminated surfaces and a 1 × 1 periodicity in the surface, containing 30 (33) to 90 (93) atoms, respectively. The use of symmetric slabs cancels out dipole moments, which can occur in nonsymmetric slabs. The vacuum layer is 15 Å thick for α-Fe_2_O_3_ films with strains up to ±6% (+ for tensile strain, − for compressive strain). The band gaps are calculated for different U parameters. The Coulomb repulsion is characterized by a spherically averaged Hubbard parameter U and a parameter J representing the screened exchange energy. While U depends on the spatial extension of the wave functions and on screening, J is an approximation of the Stoner exchange parameter and almost constant J ~1 eV. In our calculation the J is set to be 0.5 eV. Figure 1b shows the band gap of α-Fe_2_O_3_ bulk as a function of U parameter of Coulomb repulsion. The band gap increases with increasing U parameter. It is important to be aware of the fact that when using the LDA+U, in general, the total energy will depend on the parameters U and J. Thus, it is not meaningful to compare the total energies resulting from calculations with different U and/or J (U − J in the case of Dudarev’s approach [24]). The energy depends on the difference (U − J) introduced by Dudarev.

(1) $$E_{\mathrm{LSDA}+U}=E_{\mathrm{LSDA}}+\frac{\left(U-J\right)}{2}\sum_{\sigma }\left[\left(\sum_{m_{1}}n_{m_{1},m_{1}}^{\sigma }\right)-\left(\sum_{m_{1},m_{2}}{\hat{n}}_{m_{1},m_{2}}^{\sigma }{\hat{n}}_{m_{2},m_{1}}^{\sigma }\right)\right]$$

In Dudarev’s approach, the parameters U and J are not entered separately, only the difference (U − J) is meaningful. The more the U_eff_ (U − J) is, the more the band gap is. A larger (U − J) lowers the one-electron potential locally for a particular Fe d orbital and in turn reducing the hybridization with O atoms and enlarging the band gap. The band gap 2.22 eV with U = 5 eV is in agreement with experimentally derived 2.2 eV [5]. If the computational lattice constants are used for the spin state calculations, there is a band gap difference slightly compared with experimental one with same U and J. U = 5 eV provides a good description of the band gap and magnetic moment of Fe, while higher values of U give a larger band gap than the experimental one. Thus, in our following calculations, we use U = 5 eV.

## 3. Results and Discussion

The α-Fe_2_O_3_ is an AFM insulator [6] because of the strong superexchange interaction mediated by the O atoms. Different magnetic ordering including the A-type AFM (+ + − −) and G-type AFM (+ − + −) [34] states have been studied using the experimental observed lattice constants. A-type AFM ordering + + − − state is energy favored, which means that Fe atoms in short distance along the hexagonal axis, have opposite magnetic moment, while Fe atoms in larger distance have equal magnetic moments. The spin-density distributions of this system presented in Figure 1c, where the up- and down-spin densities are denoted by light yellow and green color, respectively, clearly indicating the AFM (ferromagnetic) coupling along the c-direction (ab-planes). The magnetic moments are well localized on the Fe ions. It is calculated to be 4.25 μ_B_ compared to an experimental value of 4.64 μ_B_ [35]. The corresponding band structure is shown in Figure 1d. Although there is some admixture of O-*p* states throughout both the filled and unfilled bands, the about 90% contribution to valence band comes from oxygen *p* states, while the occupied 3*d* levels of Fe lie around 6–7 eV below the Fermi level and at 0–3 eV below the Fermi level dominated by O 2*p* states is more pronounced. This is a consequence of the fact that the on-site Coulomb potential acts only on the Fe 3*d* states, leading to a reduction of the Fe–O hybridization which is too strong. The conduction band minimum (CBM) is mainly populated by the unoccupied 3*d* levels of Fe. In α-Fe_2_O_3_, the oxidation states of O and Fe are −2 and +3, respectively. Therefore, maghemite is a charge-transfer type of insulator rather than a Mott–Hubbard insulator, and the first excitation term should correspond to the transfer of electrons from the O^2^^−^ anions to the octahedral Fe^3^^+^ cations. The band structure of α-Fe_2_O_3_ has an almost dispersionless CBM between the M and K points in the first Brillouin zone seen from Figure 1d. This indicates that pure α-Fe_2_O_3_ has extremely heavy carrier effective masses, which will result in low mobility for the electrons. The conduction band between Γ and A is also very flat and at higher energy compared to the CBM. It implies that interlayer conduction along the *z*-direction will be severely suppressed; the conductivity is known to be highly anisotropic, being significantly higher within the layers in the ab-plane. On the other hand, the favorable *p*-*d* hybridization between the O *p* and Fe *d* states leads to dispersion feature between M and K points of valence band maximum (VBM). Therefore, holes have a lower effective mass, which would suggest a higher mobility compared to electrons.

**Figure 2:**
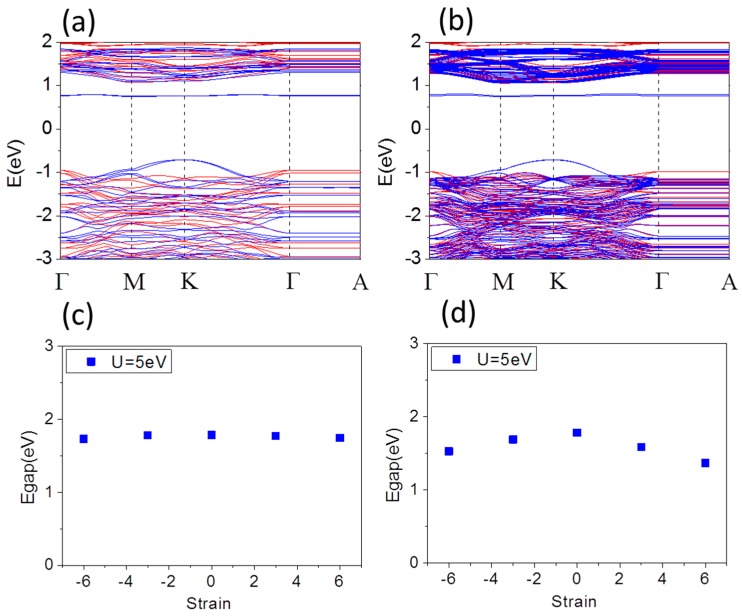
Band structure of Fe terminated α-Fe_2_O_3_: 1 × 1 × 1 film (**a**); and 1 × 1 × 3 film (**b**); for lattice constant *a* = 5.035 Å at U = 5 eV. Band gap of Fe terminated α-Fe_2_O_3_: 1 × 1 × 1 film (**c**); and 1 × 1 × 3 film (**d**), as a function of strain.

It was confirmed that the oxide film on steel after exposure to H_2_O_2_ and O_2_ environments consists mainly of hematite (α-Fe_2_O_3_) [36], although there would be Fe_3_O_4_, Fe(OH)_3_ and so on. Depending on the aqueous environment, the thickness of α-Fe_2_O_3_ thin films varies from a few to several 100 nm. There are three possible planes to terminate the bulk to form a clean (0001) surface: a single Fe layer, a double Fe-Fe layer, or an oxygen layer, as indicated in the following notation: Fe-O3-Fe-R, Fe-Fe-O3-R, and O3-Fe-Fe-R, where R represents the continuing bulk atomic stacking sequence. Under ultrahigh vacuum conditions, the experimental and theoretical evidence was consistent with a highly relaxed Fe-terminated surface, Fe-O3-Fe-R, for α-Fe_2_O_3_ [37,38]. This is the most stable surface from a stoichiometric and electrostatic perspective, as the polar oxygen anion layer is stabilized by an equal number of positive metal cations above and below [39]. For a comparative study, we have focused on two different Fe terminated (Fe-O3-Fe-R) and O terminated (O3-Fe-Fe-R) α-Fe_2_O_3_ thin film, respectively. The optimized lattice constant for 1 × 1 × 1 Fe terminated α-Fe_2_O_3_ thin film is *a* = 5.014 Å which is in agreement with experimentally derived structural parameter 5.035 Å [33]. In our study we use *a* = 5.035 Å. Figure 2a,b shows typical band structures of free standing Fe terminated α-Fe_2_O_3_ 1 × 1 × 1 and 1 × 1 × 3 thin film with thickness 1.31 nm corresponding cell with 30 atoms and 4.06 nm corresponding supercell with 90 atoms, respectively. There is almost no change in the features of the CBM because it still has Fe 3*d* character. On the Fe-terminated surface with a reduced Fe content in the top layer, the coordination of the surface Fe atom is very different from the octahedral environment in the bulk. The favorable *p*-*d* hybridization between the O *p* and Fe *d* states extends to dispersion region from M-K line to M-K-Γ of valence band maximum (VBM). The band gap is 1.78 and 1.79 eV in Figure 2a,b for Fe terminated α-Fe_2_O_3_ 1 × 1 × 1 and 1 × 1 × 3 thin film, respectively, which is smaller than that of bulk. The electronic band at the surface differs from that in the bulk mostly by a pronounced narrowing of the Fe-derived bands resulting from the reduced coordination of the Fe atoms. That does not follow the traditional rule of “the band gap of bulk is smaller than that of film” [40]. The Fe-surface moment is 3.99 (4.00) μ_B_ in 1 × 1 × 1 (1 ×1 × 3) thin film. Hence, for the Fe-terminated surface, the magnetism of the surface should not differ appreciably from that in the bulk. Moreover, the in-plane lattice constant of α-Fe_2_O_3_ thin films does not match the steel substrate exactly with a perfect coherent interface, so that compressive (− %) or tensile (+ %) strain is suggested to play an important role in affecting the electronic structure of α-Fe_2_O_3_ films. Figure 2c,d shows the band gap of 1 × 1 × 1 and 1 × 1 × 3 supercell of Fe terminated α-Fe_2_O_3_ thin film as a function of biaxial strain from compressive −6% up to tensile 6%, respectively. The band gaps of 1 × 1 × 3 supercell are significantly reduced, while the combination of the quantum confinement and surface coupling effect is likely to leads to the band gap of 1 × 1 × 1 cell decreasing slightly. Under compressive strain up to −6% the Fe-surface magnetic moment of 1 × 1 × 1 (1 × 1 × 3), supercell decreases from 3.99 (4.00) μ_B_ to 3.90 (3.80) μ_B_, while it increases slightly from 3.99 (4.00) μ_B_ to 4.04 (4.04) μ_B_ under tensile strain up to 6%.

**Figure 3:**
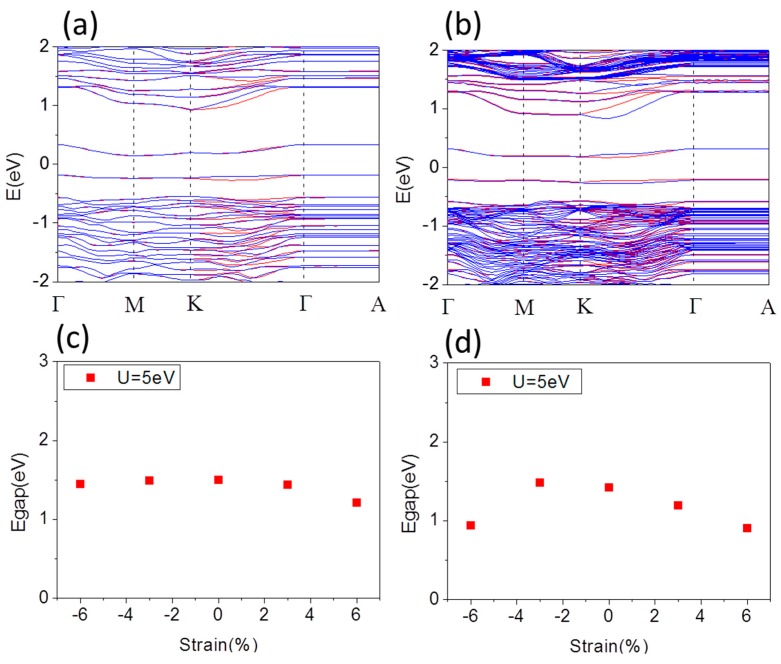
Band structure of O terminated α-Fe_2_O_3_: 1 × 1 × 1 film (**a**); and 1 × 1 × 3 film (**b**), for lattice constant *a* = 5.035 Å at U = 5 eV. Band gap of O terminated α-Fe_2_O_3_: 1 × 1 × 1 film (**c**); and 1 × 1 × 3 film (**d**), as a function of strain.

Figure 3a,b shows the typical band structure of free standing O terminated α-Fe_2_O_3_ 1 × 1 × 1 thin film with thickness of 1.37 nm corresponding cell with 33 atoms and 1 × 1 × 3 thin film with thickness of 4.12 nm corresponding supercell with 93 atoms, respectively. The band gap is 1.50 eV and 1.48 eV, respectively, which is smaller than that of bulk, even smaller than that of Fe terminated α-Fe_2_O_3_ thin film. In O terminated α-Fe_2_O_3_ thin film there is more electrons transfer from Fe to O, so that *d* Coulomb repulsion decreasing results in band gap decreasing much. The spin splitting only induces along K-Γ line. Two even bands showing spin split slightly along K-Γ line of band structure, are just below and above E_F_, which mainly comes from the *p* orbitals of O-surface atoms. In this case, the CBM becomes more dispersive than that of the pristine α-Fe_2_O_3_, indicating that O terminated α-Fe_2_O_3_ thin film may reduce the effective mass for electrons. Similarly, we investigate the electronic structure modulation of O terminated α-Fe_2_O_3_ films by strain. Figure 3c,d shows the band gap of 1 × 1 × 1 and 1 × 1 × 3 supercell of O terminated α-Fe_2_O_3_ thin film as a function of biaxial strain from compressive −6% up to tensile 6%, respectively. For both compression and tension, the band gap of 1 × 1 × 1 cell of O terminated α-Fe_2_O_3_ thin film decreases slightly, while that of 1 × 1 × 3 supercell decreases obviously. Compression increases spin splitting along K-Γ line and the CBM becomes more dispersive, correspondingly decreasing the effective mass for electrons. In contrast, it decreases along K-Γ line under tension and the CBM becomes less dispersive. Under compressive strain up to −6% the O-surface magnetic moment of 1 × 1 × 1 (1 × 1 × 3) supercell decreases from 2.68 (2.68) μ_B_ at *a* = 5.035 Å to 2.57 (2.50) μ_B_ at *a* = 4.733 Å, while it increases to 2.80 (2.80) μ_B_ at *a* = 5.337 Å under tensile strain up to 6%. Hence, for the O-terminated surface, the magnetism of the surface differs appreciably from that in the Fe-terminated surface.

The work function (*W*) is calculated as the difference between the vacuum level *E*_vacuum_ and the Fermi energy *E*_F_:

*W* = *E*_vacuum_ − *E*_F_ (2)

**Figure 4:**
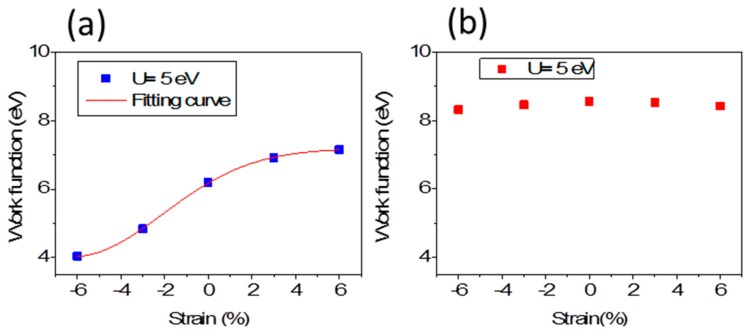
The work function of Fe terminated α-Fe_2_O_3_ 1 × 1 × 3 film (**a**); and O terminated α-Fe_2_O_3_ 1 × 1 × 3 film as a function of strain (**b**).

*W* reflects electronic energy level and is therefore related to its electrostatic potential. The strain is an effective approach to modulate the surface work function. Figure 4a,b presents the electrostatic potential (vacuum level) for the Fe-terminated and O-terminated 1 × 1 × 3 thin film changing with strain increasing, respectively. The vacuum levels (where Fermi energy is set as 0.0 eV) indicate the W relative to upper surfaces. The W for the Fe-terminated surface decreases strongly under compressive strain up to −6%, while it increases obviously under tensile strain up to 6%. For example, it is 4.85 eV under −3% strain with *a* = 4.884 Å and 4.04 eV under −6% strain with *a* = 4.733 Å, respectively, while it is 6.91 eV under 3% with *a* = 5.186 Å and 7.16 eV under 6% strain with *a* = 5.337 Å. For O-terminated 1 × 1 × 3 thin film, the W is higher than that of Fe-terminated 1 × 1 × 3 thin films and less sensitive to strain.

**Figure 5:**
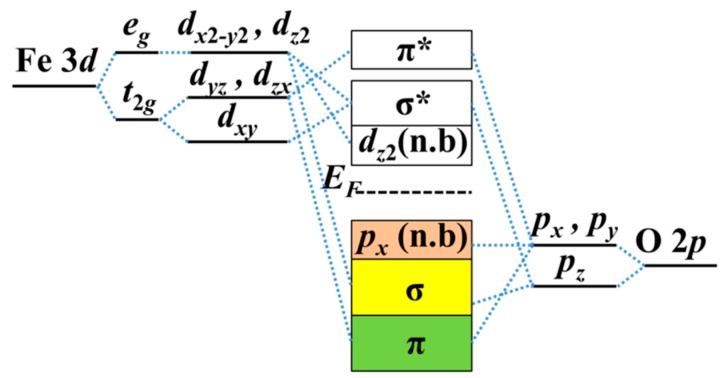
Schematic diagram of orbital energy-level of Fe_2_O_3_.

It is important to show the mechanism of band gap and W modulations by strain. In this system, the oxygen anions are arranged in an octahedral geometry around the Fe cation. In the crystal field (with ionic approach) of octahedral symmetry, two of the five *d* orbitals label *e*_g_ with point-symmetry are destabilized and pushed up in energy, and the other three *d* orbitals are stabilized and pulled down in energy with the point-symmetry label *t*_2g_. The schematic diagram of orbital energy-level of Fe_2_O_3_ is shown in Figure 5. The σ bonds are formed by head-on overlapping among Fe *d_x_*_2-*y*2_, *d_z_*_2_ and O *p_z_* orbitals, and π bonds are formed by shoulder-on overlapping among Fe *d_xy_*, *d_yz_*, *d_yz_*, and O *p_x_* (*p_y_*) orbitals. Nonbonding *p_x_* (*p_y_*) electrons occupy the VBM below *E*_F_. The O^2−^ molecular orbitals have to be higher than the Fe^3+^ orbitals in the isolated system, while below the Fermi level are the orbitals from the O atoms (occupied) and those above are from the Fe (unoccupied) in the α-Fe_2_O_3_ thin film with the ionic model.

**Figure 6:**
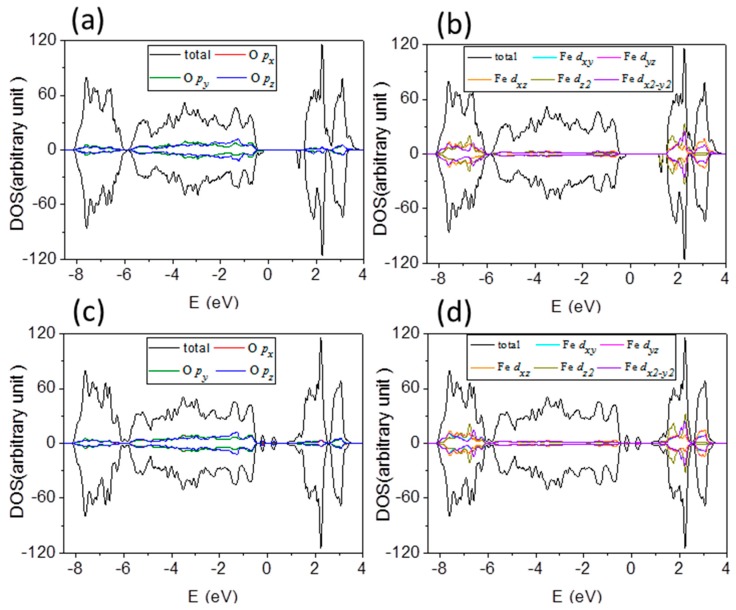
(**a**,**b**) Calculated density of states for O 2*p* and Fe 3*d* orbitals of Fe terminated α-Fe_2_O_3_ 1 × 1 × 3 film without strain, respectively, as labeled in different color; and (**c**,**d**) calculated density of states for O 2*p* and Fe 3*d* orbitals of O terminated α-Fe_2_O_3_ 1 × 1 × 3 film without strain, respectively. It is labeled in red, olive and blue for *p_x_*, *p_y_*, and *p_z_* orbitals of O atoms; and cyan, magenta, orange, dark yellow and violet for *d_xy_*, *d_yz_*, *d_xz_*, *d_z_*_2_ and *d_x_*_2_ orbitals of Fe atoms.

**Figure 7:**
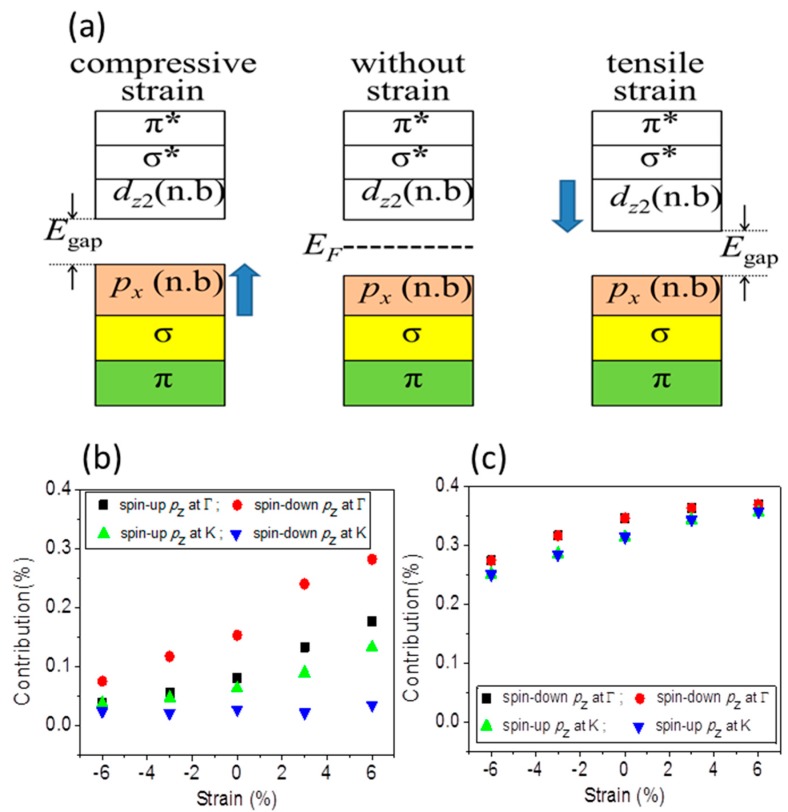
(**a**) The illustration for energy gap changing with strain. The spin polarized *p_z_* state change with increasing strain: (**b**) at K and Γ points for Fe terminated α-Fe_2_O_3_ 1 × 1 × 3 film; and (**c**) at K and Γ points for O terminated α-Fe_2_O_3_ 1 × 1 × 3 film.

Total density of states (DOS) mainly comes from O 2*p* states and Fe *d* states. It can be divided into projected DOS plots, as shown in Figure 6a,b, respectively, for O atom and Fe atom of Fe terminated 1 × 1 × 3 α-Fe_2_O_3_ films, and labeled in red, olive and blue for *p_x_*, *p_y_*, and *p_z_* orbitals of O atoms; and cyan, magenta, orange, dark yellow and violet for *d_xy_*, *d_yz_*, *d_xz_*, *d_z_*_2_ and *d_x_*_2_ orbitals of Fe atoms. One can see that DOS consist of a hybridized mixture of O and Fe states because overlap integrals between O 2*p* states and Fe *d* states are nonzero. The contributions from *p_x_* and *p_y_* states of O atoms to DOS play important role below Fermi level, while DOS above the Fermi level is dominated by Fe 3*d* states. Thus, the occupied valence band is majority O 2*p* character, and the unoccupied conduction band is majority Fe 3*d* character. Figure 6c,d presents the DOS of O and Fe atoms of O terminated α-Fe_2_O_3_ films, which reflect *p_x_*, *p_y_*, and *p_z_* states of O atoms below *E*_F_, and Fe 3*d* states are more pronounced above *E*_F_. One can see that it is a charge-transfer type of insulator rather than a Mott–Hubbard insulator, whether it is a Fe or O terminated α-Fe_2_O_3_ film. In addition to the valence state and the conduction band with significant difference, the shape of the DOS is very similar to that of Fe terminated α-Fe_2_O_3_ films. The mechanism of band gap decreasing under both compressive and tensile strains is similarly in Fe and O terminated α-Fe_2_O_3_ films. There is a consequence of the fact that the on-site Coulomb potential acts only on the Fe 3*d* states, leading to a reduction of the Fe–O hybridization which is too strong under compressive strain. That lifts the degeneracy of Fe *e*_g_ orbitals, which competes with the AFM superexchange of localized *t*_2g_ electrons. The nonbonding *p_x_* and *p_y_* states enhance under compressive and then it pushes VBM toward *E*_F_, as seen in Figure 7a for the schematic diagram of orbital energy-level of Fe_2_O_3_. Tensile strain lowers the degeneracy of Fe *e*_g_ orbitals and the antiferromagnetic superexchange interaction between *t*_2g_ spins enhances, which draw CBM with *e*_g_ (*d_z_*_2_) orbitals nearer to *E*_F_. Therefore, under both compressive and tensile strains, the band gap between the VBM and CBM decreases.

The *p_z_* states are strengthened (dropped) under tensile (compressive) strain, as seen in Figure 7b for Fe terminated α-Fe_2_O_3_ films. The *p_z_* state with spin down at K point (Figure 2a,b) is insensitive to strain, which implies the surface state, arising from localized dangling-bond states, while the bulk band, with spin up at K or Γ point, arising from extended Bloch states, is very sensitive to strain. The electrons of *p_z_* state have relatively small momentum because interlayer conduction along the *z*-direction is severely suppressed from our results on the basis of very flat conduction band between Γ and A in Figure 3. With the decrease (increase) of the contribution of the *p_z_* state, the electrons with less momentum decrease (increases), while electrons of *p_x_* and *p_y_* states with higher momentum increase (decrease), so that the total wave vector and the Fermi sphere radius increase (decrease), the corresponding Fermi energy increase (decrease). Therefore, W reading from Equation (2) decreases under compressive strain and increases under tensile strain. With our results in Figure 4a, we pbtain the expression of fitting curve for W,

(3) $$W\;=\;7.18\;-\;3.15e^{-0.03{\left(x+6.18\;\right)}^{2}}$$

where *x* presents the strain. The formula shows the rather complicated relationship between the W and strain. Previous theoretical study has not been well established the relationship between electrostatic potential (or corrosion potential) and mechanical deformation. According to previous studies of the influence of deformation on the electron gas of a metal, it is possible to qualitatively predict changes in the electrode potential [41] and hence to estimate the effect of deformation on the corrosion potential. Experimental data showed rather complicated relationship and experimental studies have shown that a simple relationship between stress and corrosion parameters does not exist [42].

For O-terminated 1 × 1 × 3 thin film, the W is higher than that of Fe-terminated 1 × 1 × 3 thin film. The *p_z_* states at Γ and K points are strengthened compared to that of Fe-terminated thin films seen from Figure 7c under different strain. The electrons of *p_z_* state are more than that of Fe-terminated thin films, whether with or without strain, and, correspondingly, more electrons have small momentum. As a result, the total wave vector and the Fermi sphere radius decreases, and the corresponding Fermi energy decreases. Therefore, W reading from Equation (2) is higher than that of Fe-terminated thin films. Although *p_z_* state at VBM is lowered under compressive strain, it is still more notable than that of Fe-terminated thin films. Moreover, the Fe–O hybridization is stronger than that of Fe-terminated thin film, so electrons are difficult to escape from the surface.

The work function is highly sensitive to surface termination. In particular, the Fe-terminated film significantly drags down work functions under compressive strain. Our results reply that compressive strain lowers the corrosion potential and leads to corrosion easily because the work function is closely related to the corrosion potential and a sensitive parameter to the corrosion behavior. Especially, the W of O-terminated film changes slightly under both compressive and tensile strains, which indicate that strains could not lower the corrosion potential much. It is confirmed that metals are protected from corrosion by the formation of “passive” oxide films.

## 4. Conclusions

In conclusion, we have carried out a systematic study of electronic properties of both Fe and O terminated α-Fe_2_O_3_ thin films under strain. Our calculations show that the band gap of α-Fe_2_O_3_ thins film may overall change with either compressive or tensile strain, gaps can survive for strains up to ±6%. These results unequivocally demonstrate that the electronic properties of both Fe and O terminated α-Fe_2_O_3_ thin films are modified by the external strains. The band gap of O terminated α-Fe_2_O_3_ thin film decreases strongly compared with that of Fe terminated α-Fe_2_O_3_ thin film. More nonbonding *p_x_* and *p_y_* orbitals at VBM account for a pronounced narrowing band gap under compressive strain, while nonbonding *d_z_*_2_ orbitals above Fermi level lead to the pronounced narrowing band gap under tensile strain. The strain is an effective approach to modulate the surface work function. Especially *p_z_* orbital, arising from extended Bloch states, is very sensitive to strain, which plays an important role for work function changing. In Fe-terminated 1 × 1 × 3 thin film, compressive strain lowers the corrosion potential and leads to corrosion easily. In particular, the work function in O terminated films is insensitive to strain because *p_z_* orbitals are less sensitive to strain than that of Fe termination films. These results have significant practical implications, which can be very useful for explaining the effect of strain, future experimental studies and fundamentally understand the corrosion behavior.

## References

[1] Ahn C.H., Triscone J.M., Mannhart J. (2003). Electric field effect in correlated oxidesystems. Nature.

[2] Rödl C., Fuchs F., Furthmüller J., Bechstedt F. (2009). Quasiparticle band structures of the antiferromagnetic transition-metal oxides MnO, FeO, CoO, and NiO. Phys. Rev. B.

[3] Jiang H., Gomez-Abal R.I., Rinke P., Scheffler M. (2009). Localized and Itinerant States in Lanthanide Oxides United by GW@LDA+U. Phys. Rev. Lett..

[4] Jiang H., Gomez-Abal R.I., Rinke P., Scheffler M. (2010). First-principles modeling of localized d states with the GW@LDA+U approach. Phys. Rev. B.

[5] Mochizuki S. (1977). Electrical conductivity of α-Fe_2_O_3_. Phys. Status Solidi.

[6] Sandratskii L.M., Kübler J. (1996). First-principles LSDF study of weak ferromagnetism in Fe_2_O_3_. Europhys. Lett..

[7] Toroker M.C., Kanan D.K., Alidoust N., Isseroff L.Y., Liao P.L., Carter E.A. (2011). First principles scheme to evaluate band edge positions in potential transition metal oxide photocatalysts and photoelectrodes. Phys. Chem. Chem. Phys..

[8] Rollmann G., Entel P., Rohrbach A., Hafner J. (2005). High-pressure characteristics of α-Fe_2_O_3_ using DFT+U. Phase Transit..

[9] Wanaguru P., An J., Zhang Q. (2016). DFT+ U study of ultrathin α-Fe_2_O_3_ nanoribbons from (110) and (104) surfaces. J. Appl. Phys..

[10] Neufeld O., Toroker M.C. (2016). Play the heavy: An effective mass study for α-Fe_2_O_3_ and corundum oxides. J. Chem. Phys..

[11] Erlebach A., Kurland H.D., Grabow J., Müller F.A., Sierka M. (2015). Structure evolution of nanoparticulate Fe_2_O_3_. Nanoscale.

[12] Kiejna A., Pabisiak T. (2013). Mixed termination of hematite (α-Fe_2_O_3_) (0001) surface. J. Phys. Chem. C.

[13] Yatom N., Toroker M.C. (2016). Electronic Structure of Catalysis Intermediates by the G_0_W_0_ Approximation. Catal. Lett..

[14] Wu D., Lagally M.G., Liu F. (2011). Stabilizing Graphitic Thin Films of Wurtzite Materials by Epitaxial Strain. Phys. Rev. Lett..

[15] Hu H., Gao H.J., Liu F. (2008). Theory of Directed Nucleation of Strained Islands on Patterned Substrates. Phys. Rev. Lett..

[16] Liu Z., Wu J., Duan W., Lagally M.G., Liu F. (2010). Electronic Phase Diagram of Single-Element Silicon “Strain” Superlattices. Phys. Rev. Lett..

[17] Sander D., Ouazi S., Enders A., Gutjahr-Löser T., Stepanyuk V.S., Bazhanov D., Kirschner J. (2002). Stress, strain and magnetostriction in epitaxial films. J. Phys. Condens. Matter.

[18] Yu D., Zhang Y., Liu F. (2008). First-principles study of electronic properties of biaxially strained silicon: Effects on charge carrier mobility. Phys. Rev. B.

[19] Van der Laan D.C., Ekin J.W. (2007). Large intrinsic effect of axial strain on the critical current of high-temperature superconductors for electric power applications. Appl. Phys. Lett..

[20] Luo H., Dong C.F. (2011). Characterization of passive film on 2205 duplex stainless steel in sodium thiosulphate solution. Appl. Surf. Sci..

[21] Li W., Li D.Y. (2005). Variations of work function and corrosion behaviors of deformed copper surfaces. Appl. Surf. Sci..

[22] Rohrbach A., Hafner J., Kresse G. (2004). Ab initio study of the (0001) surfaces of hematite and chromia: Influence of strong electronic correlations. Phys. Rev. B.

[23] Mosey N.J., Liao P., Carter E.A. (2008). Rotationally invariant ab initio evaluation of Coulomb and exchange parameters for DFT+U calculations. J. Chem. Phys..

[24] Dudarev S., Botton G.A., Savrasov S.Y., Humphreys C.J., Sutton A.P. (1998). Electron-energy-loss spectra and the structural stability of nickel oxide: An LSDA+U study. Phys. Rev. B.

[25] Huda M.N., Walsh A., Yan Y., Wei S.H., Al-Jassim M.M. (2010). Electronic, structural, and magnetic effects of 3d transition metals in hematite. J. Appl. Phys..

[26] Asinimov V.I., Zaanen J., Andersen O.K. (1991). Band theory and Mott insulators: Hubbard U instead of Stoner I. Phys. Rev. B.

[27] Chen L., Yu D.C., Liu F. (2008). Magnetism in nanopatterned graphite film. Appl. Phys. Lett..

[28] Chen L., Hu H., Ouyang Y., Pan H.Z., Sun Y.Y., Liu F. (2011). Atomic chemisorption on graphene with Stone–Thrower–Wales defects. Carbon.

[29] Wang Z.F., Chen L., Liu F. (2014). Tuning Topological Edge States of Bi (111) Bilayer Film by Edge Adsorption. Nano Lett..

[30] Chen L., Wang Z.F., Liu F. (2013). Robustness of two-dimensional topological insulator states in bilayer bismuth against strain and electrical field. Phys. Rev. B.

[31] Slater J.C. (1951). A Simplification of the Hartree-Fock Method. Phys. Rev..

[32] Vosko S.H., Wilk L., Nusair M. (1980). Accurate spin-dependent electron liquid correlation energies for local spin density calculations: A critical analysis. Can. J. Phys..

[33] Finger L.W., Hazen R.M. (1980). Crystal structure and isothermal compression of Fe_2_O_3_, Cr_2_O_3_, and V_2_O_3_ to 50 kbars. J. Appl. Phys..

[34] Guo Y.Z., Clark S.J., Robertson J. (2012). Electronic and magnetic properties of Ti_2_O_3_, Cr_2_O_3_, and Fe_2_O_3_ calculated by the screened exchange hybrid density functional. J. Phys. Condens. Matter.

[35] Coey J.M.D., Sawatzky G.A. (1971). A study of hyperfine interactions in the system (Fe_1-*x*_Rh*_x_*)_2_O_3_ using the Mossbauer effect (Bonding parameters). J. Phys. C Solid State Phys..

[36] Murayama Y., Satoh T., Uchida S., Satoh Y., Nagata S., Satoh T., Wada Y., Tachibana M. (2002). Effects of hydrogen peroxide on intergranular stress corrosion cracking of stainless steel in high temperature water, (V) Characterization of oxide film on stainless steel by multilateral surface analyses. J. Nucl. Sci. Technol..

[37] Chambers S., Yi S.I. (1999). Fe termination for α-Fe_2_O_3_ (0001) as grown by oxygen-plasma-assisted molecular beam epitaxy. Surf. Sci..

[38] Thevuthasan S., Kim Y.J., Yi S.I., Chambers S.A., Morais J., Denecke R., Fadley C.S., Liu P., Kendelewicz T., Brown G.E. (1999). Surface structure of MBE-grown α-Fe_2_O_3_(0001) by intermediate-energy X-ray photoelectron diffraction. Surf. Sci..

[39] Ruberto C., Yourdshahyan Y., Lundqvist B.I. (2003). Surface properties of metastable alumina: A comparative study of κ- and α-Al_2_O_3_. Phys. Rev. B.

[40] Wang D.C., Chen L., Liu H.M., Wang X.L., Cui G.L., Zhang P.H., Zhao D.P., Ji S.H. (2015). Topological states modulation of Bi and Sb thin films by atomic adsorption. Phys. Chem. Chem. Phys..

[41] Kolodii B.I. (2000). Theoretical Investigation of the Interaction of a Deformed Metal with a Corrosion Medium. Mater. Sci..

[42] Akid R., Dmytrakh I. (1998). Influence of surface deformation and electrochemical variables on corrosion and corrosion fatigue crack development. Fatigue Fract. Eng. Mater. Struct..

